# Math and Fossils Resolve a Debate on Dinosaur Metabolism

**DOI:** 10.1371/journal.pbio.0040255

**Published:** 2006-07-11

**Authors:** Liza Gross

Of the many mysteries surrounding the life history of dinosaurs, one of the more enduring is how such gigantic organisms—some reaching 42 feet tall and weighing 90 tons—regulated their body temperature. For many years, scientists had assumed that dinosaurs, which evolved from reptiles, were also cold blooded (ectotherms), with a slow metabolism that required the sun's heat to thermoregulate. But, in the late 1960s, the notion emerged that dinosaurs, like mammals and birds, might have been warm blooded (endotherms) with relatively constant, high body temperatures that were internally regulated like their avian descendants (and mammals).

Still others argued that while most dinosaurs had a metabolism similar to contemporary reptiles, the large dinosaurs managed a higher, more-constant body temperature through thermal inertia, which is how modern alligators, Galapagos tortoises, and Komodo dragons retain heat. Thermal inertia allows the body to approach homeothermy, or constant body temperature, when the ratio of body mass to surface area is high enough. If this “inertial homeothermy” hypothesis is correct, dinosaur body temperature should increase with body size.

In a new study, James Gillooly, Andrew Allen, and Eric Charnov revisit—and resolve—this debate. The researchers used a model that provided estimates of dinosaur body temperature based on developmental growth trajectories inferred from juvenile and adult fossil bones of the same species. The model predicts that dinosaur body temperature did increase with body mass, and that large dinosaurs had body temperatures similar to those of modern birds and mammals, while smaller dinosaurs' temperatures were more like contemporary reptiles. These results suggest that the largest dinosaurs (but not the smaller ones) had relatively constant body temperatures maintained through thermal inertia.

Gillooly et al. compiled data from eight dinosaur species from the early Jurassic and late Cretaceous periods that ranged in size from 30 pounds to 28 tons. The growth trajectories, taken from the published research papers, were determined by using bone histology (microscopic study) and body size estimates to estimate the maximum growth rate and mass at the time of maximum growth. The recent availability of these data, the researchers explain, along with advances in understanding how body size and temperature affect growth, allowed them to use a novel mathematical model to estimate dinosaur body temperatures. The researchers modified the model to estimate the body temperature of each dinosaur species, based on its estimated maximum growth rate and mass at the time of maximum growth. The model shows that body temperature increases with body size for seven dinosaur species.

The model shows that dinosaur body temperature increased with body size, from roughly 77 °F at 26 pounds to 105.8 °F at 14 tons. These results, the researchers explain, suggest that the body temperatures of the smaller dinosaurs (77 °F) were close to the environmental temperature—just as occurs for modern smaller reptiles—which meant they acquired heat from external sources (in addition to the internal heat generated by metabolism). The results also suggest that body temperature rose as an individual dinosaur grew, increasing by about 37.4 °F for species weighing about 661 pounds as adults and nearly 68 °F for those reaching about 27 tons *(Apatosaurus excelsus)*. Predicted body temperature for the largest dinosaur ( *Sauroposeidon proteles* at about 60 tons) was about 118 °F—just past the limit for most animals, suggesting that body temperature may have prevented dinosaurs from becoming even bigger.

Gillooly et al. demonstrate the validity of these results by showing that the model successfully predicts documented increases in body temperature with size for existing crocodiles. Altogether, these results indicate that dinosaurs were reptiles and that their body temperature increased with body size—providing strong evidence for the inertial homeothermy hypothesis.

## 

**Figure:**
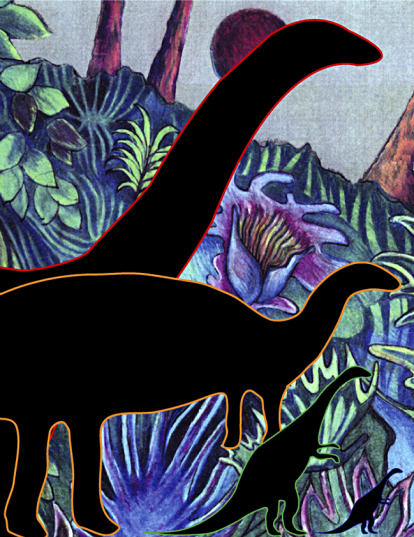
A model based on growth trajectories estimated from fossils provides evidence that dinosaurs were reptiles whose body temperatures increased with body size. A model based on growth trajectories estimated from fossils provides evidence that dinosaurs were reptiles whose body temperatures increased with body size. (Painting: Skye White)

