# Correction: Math and Fossils Resolve a Debate on Dinosaur Metabolism

**DOI:** 10.1371/journal.pbio.0040328

**Published:** 2006-10-17

**Authors:** Liza Gross

In *PLoS Biology*, volume 4, issue 8: DOI: 10.1371/journal.pbio.0040255

The figure caption is missing a credit. The caption should read as follows: A model based on growth trajectories estimated from fossils provides evidence that dinosaurs were reptiles whose body temperatures increased with body size. (Painting: Skye White).

